# Global research trends in immunotherapy for non-small cell lung cancer patients with KRAS mutations: a bibliometric analysis

**DOI:** 10.3389/fonc.2024.1385761

**Published:** 2024-05-16

**Authors:** Hanyu Shen, Chunxiao Li

**Affiliations:** ^1^ Department of Clinical Laboratory, Affiliated Huishan Hospital of Xinglin College, Nantong University, Wuxi Huishan District People’s Hospital, Wuxi, Jiangsu, China; ^2^ Department of Surgery, Wuxi Huishan No.2 People’s Hospital, Wuxi, Jiangsu, China

**Keywords:** immunotherapy, immune checkpoint inhibitors, KRAS mutations, NSCLC, bibliometric

## Abstract

**Background:**

Immunotherapy, frequently combined with conventional chemotherapy, is crucial for treating NSCLC. Kirsten rat sarcoma virus (KRAS) is a poor prognostic factor in patients with NSCLC, particularly lung adenocarcinoma, where binding of conventional inhibitors to mutated KRAS proteins is challenging. Field profiles, research hotspots, and prospects for immunotherapy for patients with NSCLC-carrying KRAS mutations were uncovered in this study.

**Methods:**

Microsoft Excel 2019, Bibliometrix, VOSviewer software, and Citespace were utilized to conduct a comprehensive scientometric analysis and understand a specific research field's knowledge base and frontiers aided by bibliometrics.

**Results:**

Between 2014 and 2023, 398 eligible documents in the English language were acquired using the WoSCC database, of which 113 and 285 were reviews and articles, respectively. The growth rate per year was 34.25 %. The most cited articles were from the United States, and China published the highest number of articles. Cancers was the journal, with increased publications in recent years. The keywords with the strongest citation bursts were analyzed using Citespace. "Immune checkpoint inhibitors," "co-occurring genomic alterations," and "KRAS" are among the research hotspots in this field.

**Conclusion:**

Using bibliometric and visual analyses, we examined immunotherapy for patients with KRAS-mutant NSCLC over the previous decade. The whole analysis showed a steady, quick increase in yearly publications in this area. Our findings will provide a roadmap for future research on the mechanisms of immunotherapy and immune checkpoint inhibitor action in treating KRAS-mutant NSCLC.

## Introduction

Lung cancer ranked first in mortality since 2020 and second in incidence, according to the most recent cancer statistics (1). Its five-year survival rate remains among the lowest despite recent advancements in early detection, molecular characterization, and development of innovative therapeutic approaches. Approximately 85% of lung cancer cases are of non-small cell lung cancer (NSCLC) (2–4). KRAS mutation is typically linked to a poor prognosis in NSCLC, with an incidence rate of 20-40% (5, 6). Treatment and medication for patients with NSCLC carrying KRAS mutation remain challenging. KRAS has four main mutational subtypes: G12C, G12V, G12D, and G12A. Among all patients with NSCLC carrying KRAS mutations, the incidences of G12C, G12V, G12D, and G12A subtypes are approximately 40%, 21%, 17%, and 8% (7–9).

Some clinical trials have reported promising results for new small-molecule inhibitors of KRAS-G12C subtype (10, 11), sotorasib (AMG510) (12), and adagrasib (MRTX849) (13), indicating their potential for use in these patients. A retrospective study showed that MRTX1133, as a non-covalent and selective KRAS-G12D inhibitor, has shown potential for tumor regression in preclinical data across multiple solid tumor models (7). Recently, the pan KRAS inhibitor BI-2865, reported by the Memorial Sloan Kettering Cancer Center and Boehringer Ingelheim (BI), has been shown to effectively inhibit the growth of various tumor cells (14). However, except for the KRAS-G12C subtype, targeted therapy for other subtypes of KRAS-mutant NSCLC is lacking. Considered undruggable, KRAS modulates the immune response in pancreatic and colorectal cancers (15). For metastatic NSCLC, immune checkpoint inhibitors (ICIs) are used as a monotherapy or combination therapy in the frontline and subsequent lines of treatment. The higher the threshold for tumor positivity for programmed death ligand 1 (PD-L1) expression, the greater the benefit. In most clinical trials examining PD-L1’s role in NSCLC, the response to ICIs has been predicted (16). Upon treatment with checkpoint therapy, clinically significant KRAS-mutated NSCLC shows a better overall survival rate than the KRAS wild-type NSCLC (17). In KRAS-mutant NSCLC, PD-L1 expression is more significant for predicting the effectiveness of ICIs compared to the other mutant types of NSCLC (18).

In 1969, Alan Pritchard introduced the concept of bibliometrics. It quantitatively examines indicators such as the volume, frequency of citations, and importance of scholarly literature. Bibliometrics gathers and processes data to thoroughly and accurately observe and characterize various patterns and phenomena. Bibliometrics aids the understanding of a specific research field’s knowledge base and frontiers (19, 20). This multi-perspective, time-phased, and dynamic technique of visual analysis of literature can automatically identify the research frontiers of the discipline and present the evolution of knowledge disciplines by displaying author networks, scholarly communication, connections between scholars, and advancements in knowledge through citation nodes and co-citation clustering. It offers a significant and workable systematic method for determining the importance of published literature. Three publications on bibliometric analyses of immunotherapy for lung cancer exist (21–23); however, to date, no bibliometric analysis on immunotherapy for NSCLC linked to KRAS mutations has been published.

In addition to examining research trends, hotspots, and boundaries from 2014 to 2023, this study aimed to conduct a bibliometric analysis in the field of immunotherapy for patients with NSCLC carrying KRAS mutations. Collaborative relationships between countries, institutions/organizations, authors, journals, references, and keywords were analyzed using Bibliometrix, VOSviewer software, Citespace, and Microsoft Excel 2019 to identify research priorities and boundaries in this area.

## Methods

### Data collection and retrieval strategy

**Figure 1:**
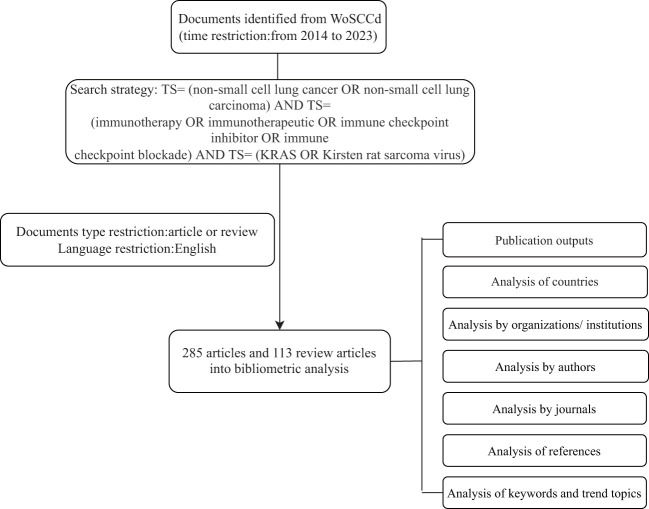
Retrieval workflow for publications related to immunotherapy for patients with KRAS mutations in NSCLC.

We searched the Web of Science Core Collection (WoSCC) database from January 1, 2014, to December 31, 2023, to obtain all publications on immunotherapy for NSCLC linked to KRAS mutations. The search strategy was as follows: TS= (non-small cell lung cancer OR non-small cell lung carcinoma) AND TS= (immunotherapy OR immunotherapeutic OR immune checkpoint inhibitor OR immune checkpoint blockade) AND TS= (KRAS OR Kirsten rat sarcoma virus). Only articles and reviews were accepted as document types, and only English was used as the language of publication. **Figure 1** illustrates the comprehensive processes of data retrieval and inclusion.

### Data analysis and visualization

Microsoft Excel 2019, Bibliometrix, VOSviewer software, and Citespace (6.2.7) were utilized to conduct a comprehensive scientometric analysis. The yearly number of publications on KRAS-mutant NSCLC immunotherapy was analyzed and plotted using Microsoft Excel 2019. We employed Bibliometrix, an R-tool available on R software (4.0.3), that generates visual representations of the results to facilitate the comprehensive scientometric analysis and statistical data. The VOSviewer 1.6.19 software was used to perform a thorough literature visualization and bibliometric analysis (24), focusing on quantifying the extent of research related to NSCLC immunotherapy across biological fields. VOSviewer was used to analyze countries, institutions, references, and keywords intuitively. CiteSpace was utilized to provide an intuitive understanding of the research bursts and evolutionary process (25, 26).

## Results

### Publication outputs

**Figure 2:**
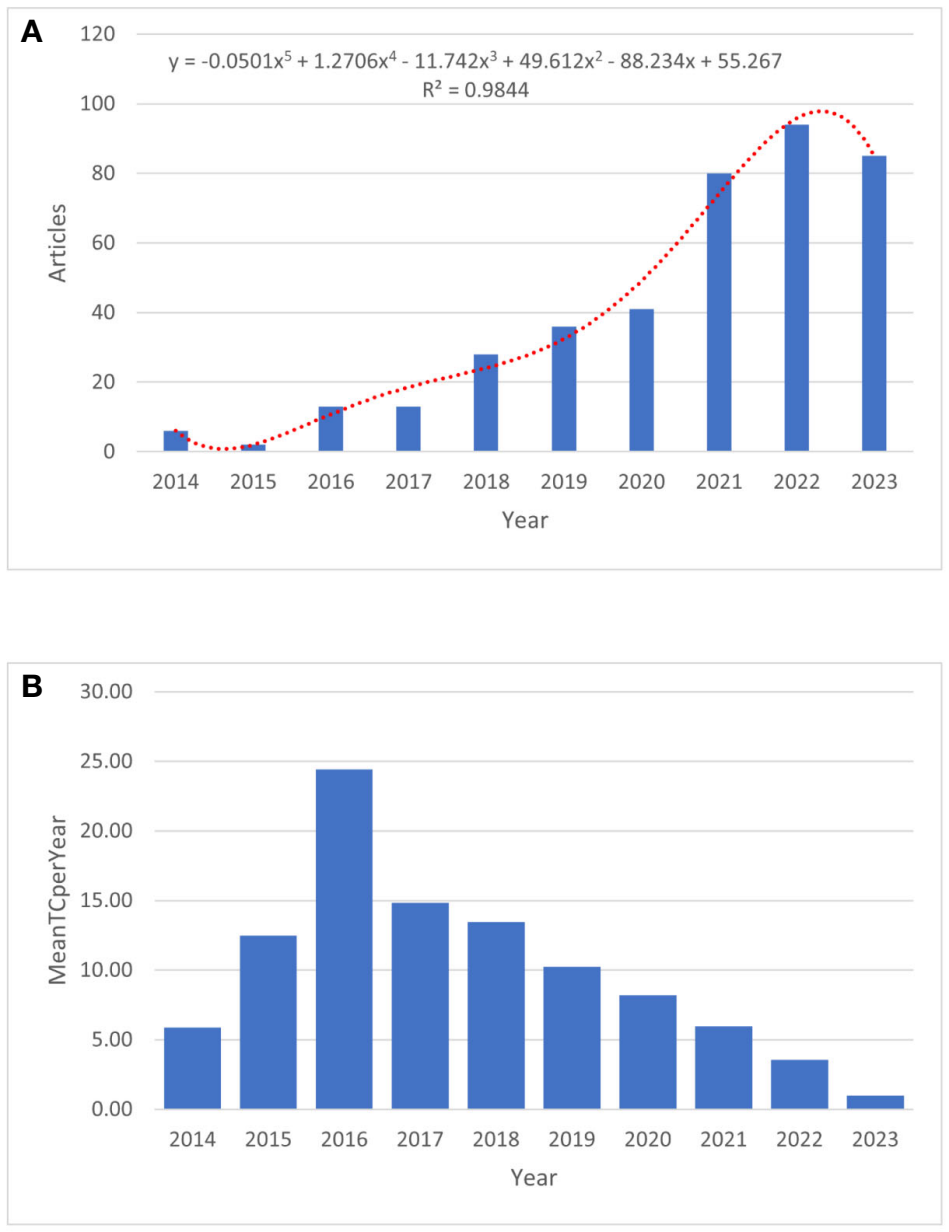
The publication trend of immunotherapy in NSCLC patients with KRAS mutation. **(A)** The bar graph displays the annual publication volume. **(B)** The annual mean total citations.

A total of 398 publications related to immunotherapy and KRAS mutation in NSCLC between 2014 and 2023 were obtained from the WOS core collection database. As shown in **Figure 2A**, with an average of 40 published papers annually, the lowest number of published papers in any given year was 2 in 2015, and the highest was 94 in 2022. A statistically significant relationship (R^2 = ^0.9844) between the number of publications and the year was obtained by fitting a mathematical function to the annual number of publications curve. The fitting curve indicated an upward trend in published articles since 2013. The number of publications on this topic has rapidly increased over the past decade, and more research opportunities exist at present. The annual mean total citations in immunotherapy for patients with NSCLC carrying KRAS mutation is shown in **Figure 2B**.

### Analysis by countries

**Figure 3:**
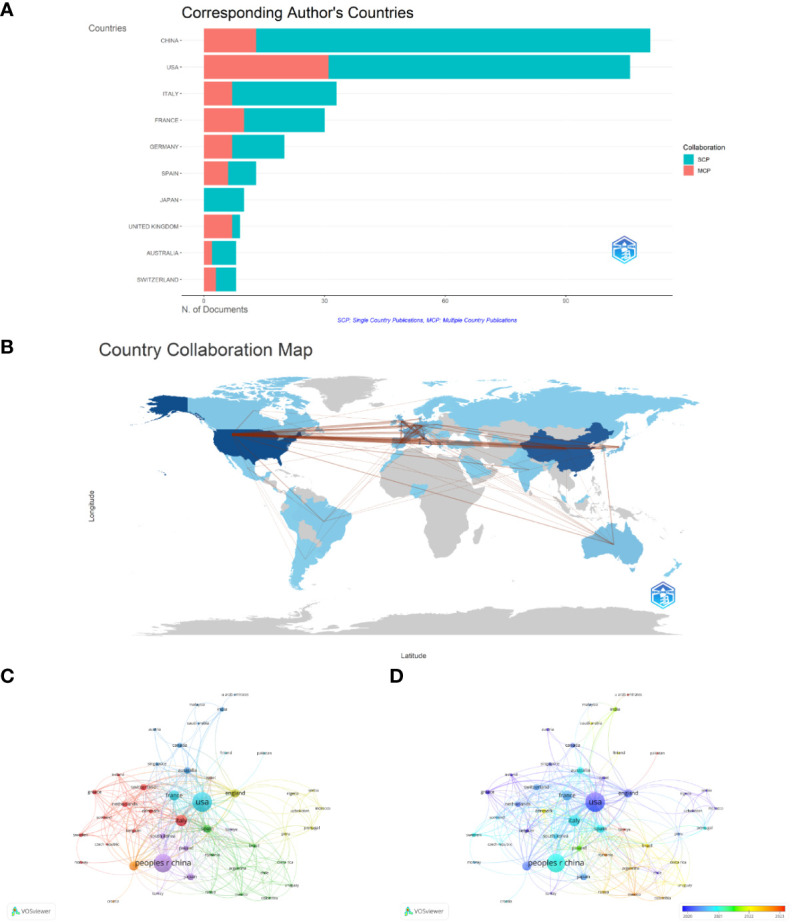
Contributions of countries. **(A)** Geographic distribution of research. **(B)** Country collaboration map. **(C)** Country/territory collaboration analysis by VOSviewer. **(D)** The time trend visualization for country co-authorship networks.

**Figure 3A** shows the geographic distribution of research on immunotherapy for patients with NSCLC carrying KRAS mutations. The countries of the top five corresponding authors were the People’s Republic of China (111, 27.9%), the United States (106, 26.6%), Italy (33, 8.3%), France (30,7.5%), and Germany (20, 5.0%). The United States has the highest number of multiple-country publications (MCP), while China has the most single-country publications (SCP). A collaborative network world map shows the collaboration between countries (**Figure 3B**). The most closely connected countries were China and the United States. A map overlay visualization of nations/regions working together on immunotherapy for patients with NSCLC carrying KRAS mutations (**Figure 3C**). **Figure 3D** displays the time trend visualization for the nation-wise co-authorship networks. The node’s color, ranging from blue to red, indicates the country’s academic activity time, while the node’s size indicates the nation’s output.

### Analysis by organizations/institutions

**Figure 4:**
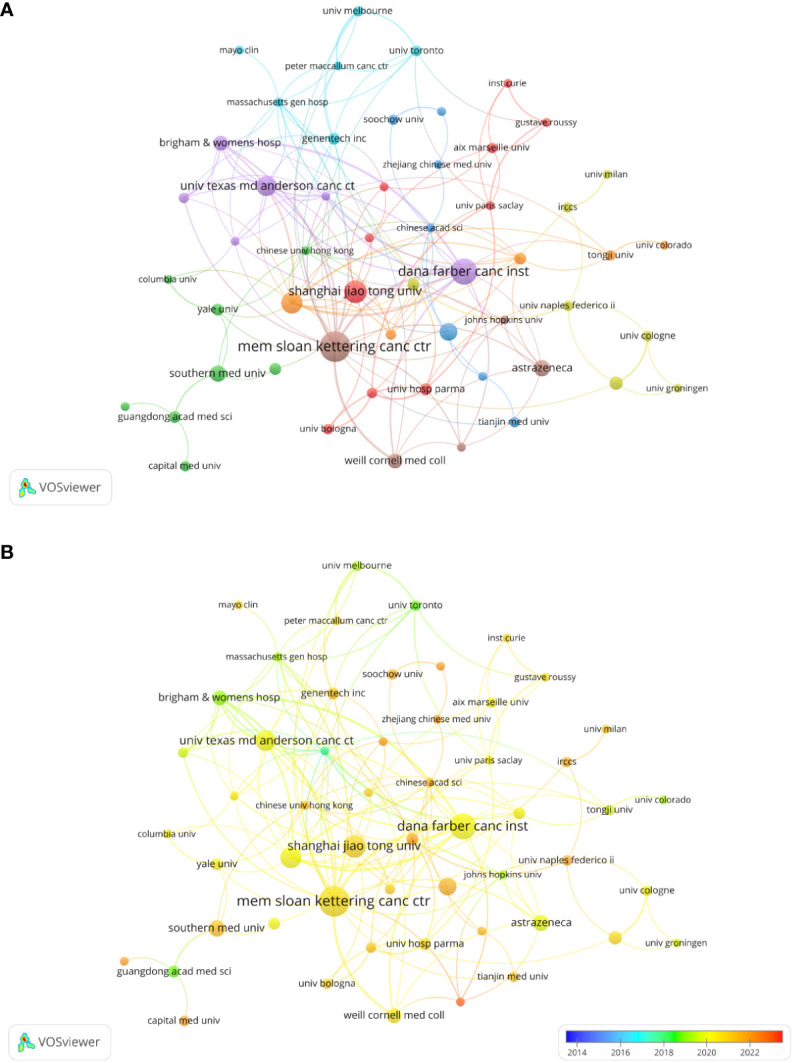
Analysis of organization/institutions. **(A)** Network visualization for institutional co-authorship analysis by VOSviewer. **(B)** Overlay map of institutional publications and average years.

In total, 1045 organizations and institutions released 398 documents. Visualization analysis included 57 organizations that met the inclusion criteria (publications > 4). The top three most productive organizations were Shanghai Jiao Tong University (14 documents), Dana-Farber Cancer Institute (17 documents), and Memorial Sloan-Kettering Cancer Center (21 documents). Six of the top ten publishing organizations were based in the United States, three in China, and one in the United Kingdom. **Table 1** shows the total yearly publications of the top ten institutions. We analyzed the co-authorship between the organizations (**Figure 4A**). A co-authorship network map of all the countries was created using VOSviewer to examine the collaboration between organizations. Excluding three institutions without connection with other organizations, all 54 top publishing institutions could be divided into eight clusters. Research from organizations in China, such as Nanjing University, Zhejiang University of Traditional Chinese Medicine, and Capital Medical University, is relatively new according to the overlay visualization maps of organizations (**Figure 4B**).

**Table 1:** The top 10 most publishing institutions according to publications.

Rank	Organization	Documents	Citations	Country
1	Memorial Sloan-Kettering Cancer Center	21	2523	USA
2	Dana-Farber Cancer Institute	17	2128	USA
3	Shanghai Jiao Tong University	14	233	China
4	Harvard Medical School	13	464	USA
5	UT MD Anderson Cancer Center	12	1596	USA
6	Chinese Academy Medical Science & Peking Union Medical College	10	255	China
7	AstraZeneca	9	446	UK
8	Southern Medical University	9	683	China
9	Brigham and Women’s Hospital	8	1667	USA
10	Weill Cornell Medical College	8	708	USA

### Analysis by authors

**Figure 5:**
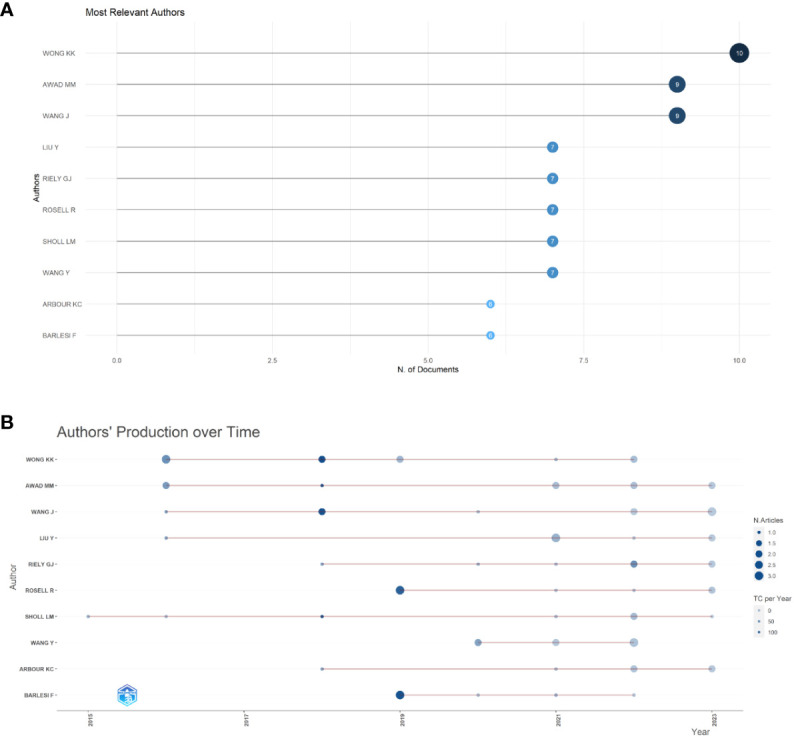
Analysis of authors’ output. **(A)** Top 10 productive authors in immunotherapy for NSCLC patients with KRAS mutations. **(B)** Authors’ production over past decade.

In total, 3524 authors contributed to this field over the past decade. Among these, the top three authors are shown in **Figure 5A**. The top author with the most publications was Kwok-Kin Wong. Studies focus on STK11/LKB1 mutations with the immune microenvironment of NSCLC harboring KRAS mutations, and the impact of TSC1/TSC2 deficiency on immune checkpoint blockade in NSCLC (27–29). **Figure 5B** shows the authors’ production over the past decade. The authors who have published more articles in the past two years are Mark M Awad (30) and Jing Wang (31).

### Analysis by journals

**Figure 6:**
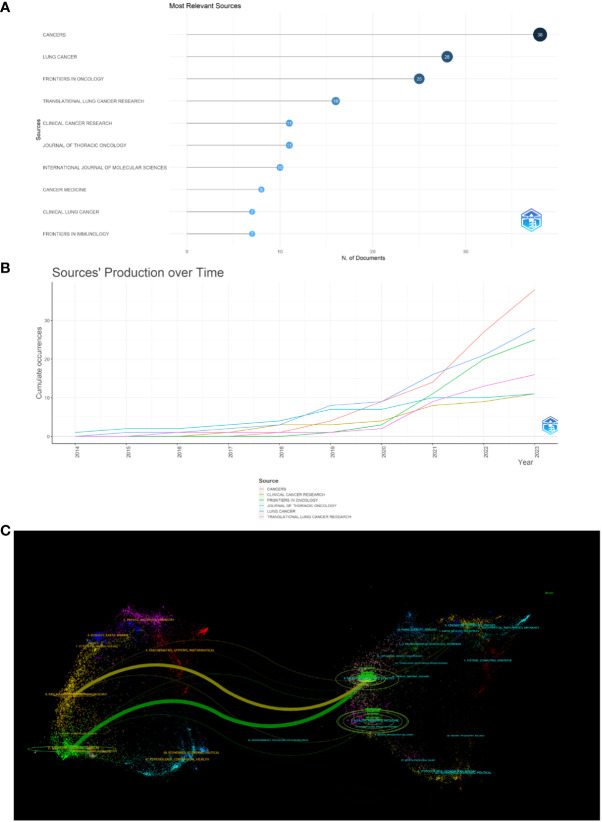
Analysis of journals. **(A)** Most relevant sources. **(B)** Sources’ production from 2014 to 2023. **(C)** Overlay map of journals.

Papers on immunotherapy for patients with NSCLC carrying KRAS mutations were published in 143 journals. As shown in **Figure 6A**, the five leading journals in publications were *Cancers* (N = 38), *Lung Cancer* (N = 28), *Frontiers in Oncology* (N = 25), *Translational Lung Cancer Research* (N = 16), *Clinical Cancer Research* (N = 11), *Journal of Thoracic Oncology* (N = 11), *International Journal of Molecular Sciences* (N = 10), *Cancer Medicine* (N = 8), *Clinical Lung Cancer* (N = 7), and Frontiers in Immunology (N = 7). More detailed journal information is listed in **Table 2**, according to the “2022 Incites Journal Citation Report,” journal citation report (JCR) quartile and impact factor (IF) were defined. **Figure 6B** shows the sources’ production over time. The publication volume of *Cancers* has increased significantly in recent years. The primary citation lines are indicated in orange in **Figure 6C**, which presents an overlay map of journals showing the citation trajectory of interdisciplinary collaboration. The studies published in molecular biology and immunology journals mainly cited reports published in journals on molecular biology and genetics. The journal’s discipline in the figure is indicated by the label on the right, where the cited paper was published. As a journal publishes more papers, the vertical axis of the ellipse in the figure on the left extends, while the horizontal axis increases as the number of authors increases.

**Table 2:** The top 10 most productive journals according to publications.

Rank	Sources	Articles	IF (2022)	JCR
1	*Cancers*	38	5.2	Q1
2	*Lung Cancer*	28	5.3	Q2
3	*Frontiers in Oncology*	25	4.7	Q2
4	*Translational Lung Cancer Research*	16	4.0	Q2
5	*Clinical Cancer Research*	11	11.5	Q1
6	*Journal of Thoracic Oncology*	11	20.4	Q1
7	*International Journal of Molecular Sciences*	10	5.6	Q2
8	*Cancer Medicine*	8	4.0	Q2
9	*Clinical Lung Cancer*	7	3.6	Q2
10	*Frontiers in Immunology*	7	7.3	Q1

### Analysis of references

**Figure 7:**
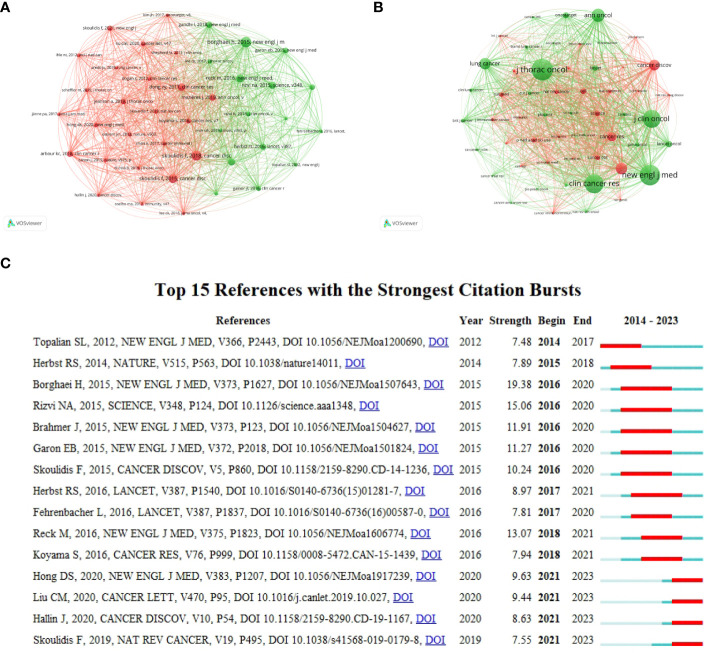
Analysis of references. **(A)** co-cited references. **(B)** co-cited sources. **(C)** Top 15 references with the strongest citation bursts.

Co-cited references and co-cited sources analyzed using VOSviewer are shown in **Figures 7A, B**. It is divided into two clusters, and most references cited in the documents were published in *Journal of Thoracic Oncology*, *The New England Journal of Medicine*, *Clinical Cancer Research*, *Journal of Clinical Oncology*, and *Annals of Oncology*. Citation bursts are a useful indicator for tracking the interest of academics in a field over time. **Figure 7C** shows the top 15 references with the strongest citation bursts from our study, as determined using CiteSpace. The article titled “Nivolumab versus Docetaxel in Advanced Nonsquamous Non-Small-Cell Lung Cancer” published in 2015” (32), ranked first in terms of strength, with a value of 19.38. The citation bursts for articles authored by Hong DS, Liu CM, Hallin J, and Skoulidis F have been continuously cited from 2021 to 2023 (33–36), demonstrating ongoing consideration for the authors’ research direction.

### Analysis of keywords and trend topics

**Figure 8:**
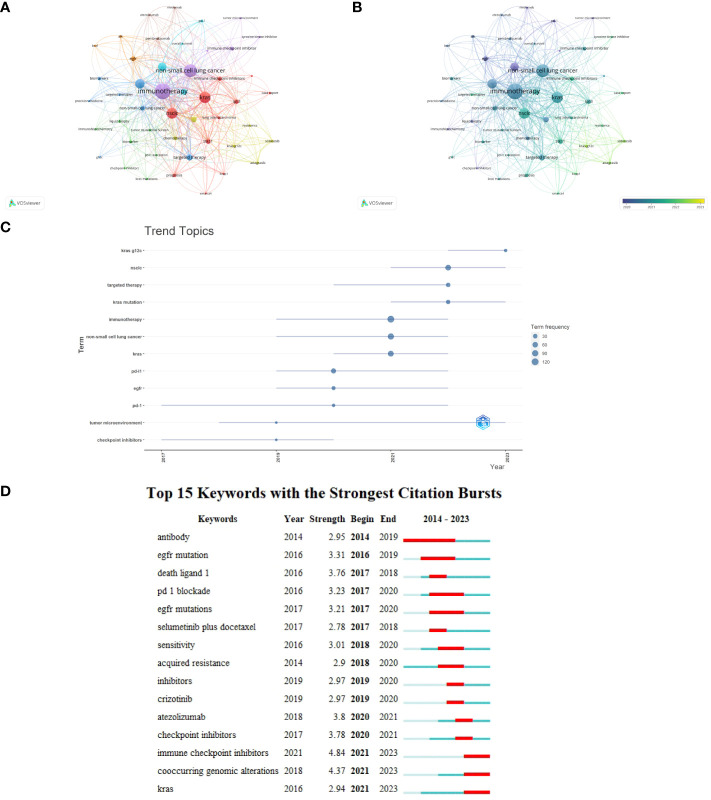
Analysis of keywords. **(A)** Clusters for keywords co-occurrence by VOSviewer. **(B)** Overlay visualization maps of keywords. **(C)** Trend Topics through Bibliometrix. **(D)** Top 15 keywords with the strongest citation bursts.

After merging synonyms and removing superfluous terms, a visualization map of keywords was generated using the VOSviewer program. Consequently, 812 keywords were identified, including 47 terms with five or more occurrences. Eight clusters were formed (**Figure 8A**). Overlay visualization maps showed around 2020, researchers focused more on immunotherapy for NSCLC patients with KRAS mutations, and in the past two years, they have focused on targeted drugs such as KRAS-G12C-related targeted therapy research. (**Figure 8B**). The timeline view of keywords intuitively showed the changing trend of research topics over time (**Figure 8C**). ICIs, tumor microenvironment, and PD-1 were early research subjects in this field. KRAS-G12C, NSCLC, and target therapy, located at the far right of this line, are new research trends in this field. **Figure 8D** shows the evolution of research hotspots in the past decade. As shown in the figure, “immune checkpoint inhibitors,” “co-occurring genomic alterations,” and “KRAS” were the keywords with the strongest citation bursts from 2021 to 2023.

## Discussion

### Overview of the results

This study identified documents on immunotherapy for patients with NSCLC carrying KRAS mutations from 2014 to 2023 by conducting a thorough literature review based on the WoSCC database. The present scientometric study included 398 English-language publications from 143 journals. The annual growth rate is 34.25%. The results indicate an increasing trend in the number of publications, suggesting growing interest in this topic, particularly due to recent advancements in the field of immunotherapy for patients carrying KRAS mutations. The annual global publication is an intuitive measure of the progress made in a particular field of study. Over the last decade, research on immunotherapy for patients with NSCLC who carry KRAS mutations has increased. Between 2020 and 2021, there was a 48.75% growth rate in publications related to immunotherapy for patients with NSCLC who carry KRAS mutations. This implies that new clinical applications have emerged along with the uncovering of the underlying mechanisms of immunotherapies for these patients. More researchers are investing time and attention in this field, as evidenced by the sharp increase in publications and citations. In the upcoming years, the attention rate of immunotherapy for KRAS-mutant NSCLC will increase, particularly for treating patients with advanced NSCLC.

Although China has the highest number of publications, its citation count is not as high as that of the USA. More than half of the reports were from the United States, indicating its outstanding position in this field. The United States is home to some of the best cancer research facilities and medical facilities worldwide, such as the Memorial Sloan-Kettering Cancer Center, Dana-Farber Cancer Institute, and UT MD Anderson Cancer Center. These institutions conduct basic research to explore the root causes of cancer and strive to develop new diagnostic tools, treatment methods, and prevention strategies. Through keyword analysis, it was found that researchers have been paying more attention to KRAS-G12C, NSCLC, and target therapy in the past two years. This might be related to the higher prevalence of KRAS-G12C mutation in NSCLC patients among KRAS mutant cancer patients. A recent analysis of a series of patients with metastatic KRAS-mutant NSCLC showed that G12C has a higher tumor mutation burden (TMB) and PD-L1 expression, which may be sensitive to immunotherapy.

### Status of research

Targeted therapy for patients with advanced KRAS mutations remains challenging. According to existing guidelines, immunotherapy targeting PD1 and PD-L1 is the best treatment method (37, 38). KRAS mutation is associated with the efficacy of ICIs (39). A real-world retrospective study revealed that the effectiveness of first-line ICIs, either alone or in combination with chemotherapy, did not differ among patients with different isoforms of KRAS mutations. Patients with KRAS-G12D and KRAS-G12A mutations showed a shorter median progression-free survival (mPFS), which did not reach statistical significance (40). The intermediate mechanism of immunotherapy for various KRAS mutation subtypes merits investigation. Recent clinical studies have shown that patients carrying KRAS mutations respond well to ICI treatment after chemoradiotherapy (CRT) treatment (41). In late-stage NSCLC, monotherapy with KRAS^wt^ resulted in poorer OS compared to KRAS-mutant patients (42). An increasing threshold for tumor positivity for PD-L1 expression was associated with a greater benefit. A trend was obtained toward a correlation between PD-L1 expression in tumor cells and the objective response rate (ORR) and progression-free survival (PFS). A recent study conducted by Wang found higher expression of immunotherapy indicators (43) (PD1, PD-L1, PD-L2, CYT, and GEP) in the KRAS-G12V and KRAS-G12D subtypes.

AMG510 and MRTX849 have been approved for treating patients with advanced NSCLC who carry G12C mutation (44). Patients with KRAS-G12C mutation in NSCLC received first-line treatment with the KRAS^G12Ci^ MRTX849 in combination with pembrolizumab, resulting in a disease control rate (DCR) of 100% (45). KRAS^G12Ci^ can reverse the immunosuppressive environment and make cancer cells sensitive to immunotherapies such as ICIs (46). However, KRAS^G12Ci^ rapidly develops resistance, as evidenced in clinical trials (47), and less than 50% of patients benefit from KRAS^G12Ci^. The combination of KRAS^G12Di^ MRTX1133 and immune checkpoint inhibitors can activate the FAS pathway, continuously inhibit tumor growth, enhance the ability to clear cancer cells, and improve survival outcomes (48). This suggests that immunotherapy’s effectiveness can be enhanced. Significant differences in TMB levels were observed among the four KRAS subtypes, with the KRAS-G12D subtype having the lowest TMB (49). In patients carrying KRAS mutations, the abundance of different immune cells varies across different subtypes. Th cells can spontaneously bind to PD-L1, blocking the anti-tumor immune response mediated by T cells. Outcomes of immunotherapy for patients with KRAS mutations are affected by several internal and external factors.

The co-mutation status is strongly suggested to affect the effectiveness of immunotherapy (50). KRAS mutations often co-exist with other mutations. A study by the National Network Genomic Medicine (NNGM) Lung Cancer Collaborator Group found that patients (PD-L1 ≥50%) carrying KRAS mutations, especially G12C and TP53 co-mutations, have better survival after receiving treatment with pembrolizumab (51–53). The co-existence of STK11 mutation and KRAS-G12C mutation can lead to poorer immune checkpoint inhibitor treatment efficacy in patients with LUAD (29). A recent study by UT MD Anderson Cancer Center found that when KEAP1, SMARCA4, and CDKN2A co-mutate with KRAS-G12C, KRAS^G12Ci^ monotherapy is ineffective in treating patients with advanced lung cancer (54). TP53 and STK11 are two common co-mutations of KRAS-G12D, and KRAS-G12D/STK11 co-mutations may be negatively correlated biomarkers for immunotherapy (55). The loss of function mutation of NKX2-1/CDKN2A can induce tumor development in patients with KRAS-G12D mutation in lung mucinous adenocarcinoma (7). For patient stratification and treatment option selection, other biological factors, such as TMB, co-mutation status, and KRAS mutation subtypes, need to be considered in addition to PD-L1 status.

### Limitations

First, this research was restricted to relying on data from the WoSCC database and did not consider literature from other databases. The analysis was limited to documents in the English language, publication type of article, and reviews. While bibliometric analyses are valuable for identifying trends and hotspots in a field, they inherently focus on quantitative metrics, such as publication volume and citation counts. Software limitations may have prevented the modification of case formats and abbreviations; inevitably, this led to the partial inclusion of articles. Thus, this study may not have fully captured the quality of research, the clinical applicability of findings, or the nuances of scientific debate within the field. Second, due to the regular updation of the database, there was a certain lag in the data obtained, such as the number of articles and citations. Finally, the dearth of keywords or abstracts may increase the chances of being excluded due to poor discoverability.

## Conclusion

Using bibliometric and visual analyses, we examined immunotherapy for patients with KRAS-mutant NSCLC over the previous decade. The whole analysis showed a steady, quick increase in yearly publications in this area. In terms of research, the United States is currently in the lead. Bibliometric analysis of keywords revealed researchers focus on the survival of certain patients carrying KRAS mutations, targeted therapy combined with immunotherapy is a highly effective therapy for patient survival, but it is also necessary to monitor whether patients have target co-mutations. However, the nature of this intermediate mechanism remains unclear. Future international cooperation between nations, organizations, and writers is expected to hasten the development of immunotherapy targeting KRAS mutations in NSCLC in conjunction with additional treatment trials. This can aid early disease diagnosis and offer useful approaches to both treatment and prevention.

## Data availability statement

The raw data supporting the conclusions of this article will be made available by the authors, without undue reservation.

## Author contributions

HS: Conceptualization, Funding acquisition, Investigation, Methodology, Software, Validation, Writing – original draft. CL: Investigation, Methodology, Software, Validation, Visualization, Writing – review & editing.
